# Stereotactic Body Radiotherapy for Oligometastatic Disease in Non-small Cell Lung Cancer

**DOI:** 10.3389/fonc.2019.01219

**Published:** 2019-11-12

**Authors:** Caryn Wujanto, Balamurugan Vellayappan, Shankar Siva, Alexander V. Louie, Matthias Guckenberger, Ben J. Slotman, Hiroshi Onishi, Yasushi Nagata, Mitchell Liu, Simon S. Lo

**Affiliations:** ^1^Department of Radiation Oncology, National University Cancer Institute Singapore, National University Health System, Singapore, Singapore; ^2^Division of Radiation Oncology and Cancer Imaging, Peter MacCallum Cancer Centre, University of Melbourne, Melbourne, VIC, Australia; ^3^Department of Radiation Oncology, Sunnybrook Health Sciences Centre, The University of Toronto, Toronto, ON, Canada; ^4^Department for Radiation Oncology, University Hospital Zurich, Zurich, Switzerland; ^5^Department of Radiation Oncology, Amsterdam University Medical Centers, Vrije Universiteit Amsterdam, Amsterdam, Netherlands; ^6^Department of Radiology, University of Yamanashi, Kofu, Japan; ^7^Department of Radiation Oncology, Graduate School of Biomedical Sciences, Hiroshima University, Hiroshima, Japan; ^8^Department of Radiation Oncology, British Columbia Cancer Agency, Vancouver Centre, Vancouver, BC, Canada; ^9^Department of Radiation Oncology, University of Washington School of Medicine, Seattle, WA, United States

**Keywords:** stereotactic body radiotherapy, non-small cell lung cancer, lung cancer, oligometastases, oligometastatic disease

## Abstract

Metastatic non-small cell lung cancer (NSCLC) is associated with a limited survival when treated with palliative intent platinum-based chemotherapy alone. Recent advances in imaging and therapeutic strategy have identified a subset of patients with limited metastases who may benefit from early local ablative therapy with either surgery or radiotherapy, in addition to standard treatment. Stereotactic body radiotherapy (SBRT) is increasingly used in the treatment of extra-cranial oligometastatic NSCLC (OM-NSCLC) due its non-invasive conduct and ability to deliver high doses. Clinical evidence supporting the use of SBRT in OM-NSCLC is emerging and consistently demonstrates significant benefit in local control and progression-free survival. Here, we discuss the definition of oligometastases (OM), review current available data on SBRT treatment in extra-cranial OM-NSCLC including evidence for site-specific SBRT in lung, liver, and adrenal metastases.

## Introduction

Lung cancer continues to be the leading cause of cancer death in many countries (1). Unfortunately, about two-thirds of non-small cell lung cancer (NSCLC) patients present with metastatic disease (Stage IV) at diagnosis and are considered incurable (2). For these patients, systemic therapy continues to be the mainstay of treatment. However, with conventional chemotherapy alone, the median survival hovers around 10 months, and long-term survival is unlikely (3). There can be considerable heterogeneity within stage IV classification, with a sub-group of stage IV patients (especially those with low-volume metastatic disease) having prolonged survival. This led to the 8th edition of American Joint Committee on Cancer (AJCC) to further categorize stage IV. In particular, patients with a single extra-thoracic metastasis was classified as M1b (Stage IVA), as opposed to patients with multiple lesions in one or multiple organs (M1c, Stage IVB) (4). Precisely classifying these patients improves the prognostic value and in doing so, will help guide treatment; in particular, identifying patients with OM-NSCLC who may warrant aggressive management of the primary tumor, as well as the metastatic sites.

The term “oligometastatic” disease has been used commonly (and sometimes loosely) in the cancer literature ever since 1995. Hellman and Weichselbaum were the first to introduce this concept of OM disease, which represented an intermediate state in the spectrum between locally confined and widely metastatic cancer (5, 6). They proposed that the process of metastatic disease occurs in a step-wise manner, and patients with limited disease should be managed aggressively. In more recent years, advances in systemic/targeted therapy may render a greater proportion of patients with upfront widely metastatic disease to a state of limited volume metastatic disease. In these patients, aggressive management of drug-resistant clones may improve cancer outcomes.

Surgical metastasectomy was initially the only way to radically manage these patients (7). With the advent of intra-cranial stereotactic radiosurgery, high doses of ablative radiation delivered over a limited number of fractions were seen to be as effective as surgical resection (8, 9). Advances in imaging, treatment delivery and patient immobilization now allow us to perform ablative radiation to extra-cranial sites in the form of stereotactic body radiotherapy (SBRT) (10). SBRT has an advantage over surgical metastasectomy in that it is non-invasive, well-tolerated and has fewer interruptions to systemic therapy.

In this mini-review, we will discuss the definitions of OM disease (in the context of NSCLC), patient selection, prognostic factors as well as completed and ongoing trials to support the use of SBRT for OM-NSCLC.

## Incidence and Definition of Oligometastatic Cancer

To date, there is no universal definition of what constitutes OM with regards to the number of lesions or sites involved. The most accepted number of metastatic lesions is considered to be 5 or less (with up to 3 metastases in an organ) (11–14).

As definitions of OM vary from study to study, it is hard to estimate the exact incidence of OM in NSCLC at diagnosis. Moreover, the routine use of staging FDG/PET-CT scan and MRI brain imaging may increase the incidence of OM, due to increased sensitivity compared to older imaging modalities. The International Association for the Study of Lung Cancer (IASLC) Lung Cancer staging project found that 225 out of 1,025 (22%) patients had synchronous single metastatic lesion at diagnosis; this group of patients had a better prognosis compared to patients with metastases in multiple organs (15). In another study by Parikh et al., 26% of patients had 5 or fewer metastases at diagnosis, and half of these patients only had 1 metastases (16).

In terms of classifying oligometastatic cancer, there are three possible scenarios:

1. Synchronous oligometastatic disease: Patients who present with up to 5 metastatic lesions (in one or a few organs) at first or within 6 months of diagnosis. These typically occur in the brain, lung parenchyma, liver or bone (15).
2. Oligo-residual (or oligo-persistent) disease: Widely metastatic disease (>5) at diagnosis, which has responded well to systemic therapy (i.e., complete response), with the remaining lesions (up to 5) amenable to radical local therapy (e.g., surgery, SBRT, RFA) (17).
3. Metachronous (or oligo-recurrence): Patients who had been treated with curative intent, and then present with limited sites of metastatic disease (up to 5) after an interval of stable disease (18).

As oligoprogression is a biologically distinct entity whereby patients with upfront widespread metastases progress, in a limited number of sites, after initially achieving stable disease or partial response, we have not included it in this definition. It is possible that patients with oligoprogression have a worse prognosis compared to the above scenarios.

## Choice of Local Therapy: Between Surgery, SBRT, and Radiofrequency Ablation

Selecting the most effective method for local treatment of oligometastases requires thoughtful considerations. Patient-related factors (e.g., age, performance status and organ function, patient preferences), tumor-related factors (e.g., location, size, proximity to vessels or nearby critical organs) and treatment-related factors (e.g., availability of expertise, cost, and waiting list) have to be taken into account.

In the latest National Comprehensive Cancer Network (NCCN) guideline for stage IVA NSCLC, definitive RT to OM, with particular mention of SBRT, is recommended as an appropriate option in suitable patients with good performance status provided it can be delivered safely (19). This reflects a growing trend and clinical evidence supporting the use of SBRT for OM. A survey of 1,007 radiation oncologists from 43 countries published by Lewis et al. in 2017 reported that 83% have been using SBRT for extracranial OM since 2005 (with over 30% since 2010) with treatment response and durability as the main reason for choosing SBRT (20). The survey reported the most common treated organs were lung, liver, and spine (90, 75, and 70%, respectively).

There are no head-to-head studies comparing surgery, SBRT, and RFA. In liver metastases, SBRT is superior to RFA in treating larger lesions >3 cm, or for lesions near blood vessels where there can be a heat-sink effect with RFA (21, 22). Widder et al. retrospectively analyzed 110 patients with pulmonary OM who were offered surgery as first line treatment for OM and SBRT if they were unsuitable for surgery (23). Although SBRT was offered as an alternative option, OS and local control rates were comparable between the two groups. As such, due to its non-invasive conduct and ability to deliver highly conformal high dose radiotherapy, SBRT has been increasingly used to target OM lesions especially for patients with technically unresectable lesions or those who are unfit for surgery.

## Prognostic Factors and Patient Selection

Patient selection is not only important to ensure the safe delivery of SBRT but also has prognostic significance (24). Several previous studies have attempted to streamline patient selection through identifying prognostic factors.

In a retrospective cohort study involving 186 patients, ECOG performance status >2, higher nodal-status (N2-3), squamous histology and metastases to multiple organs were associated with a worse prognosis (16). Ashworth and colleagues performed an individual patient meta-analysis using data from 757 patients treated curatively at the primary site, and with up to 5 metastatic lesions, treated radically with local therapies such as surgical resection, SBRT, high-dose radical RT (25). Surgery was the most commonly used treatment for the primary site (83.9%) and the metastatic sites (62.3%). The median survival of these patients was 26 months, and approximately a third survived 5 years. Key findings from this study are that patients with metachronous metastases, lower N status and adenocarcinoma histology were predicted to have longer OS. The authors proposed stratifying patients into three risk groups: low-risk (metachronous metastases, 5-year OS 47.8%), intermediate-risk (synchronous metastases with N0 disease, 5-year OS 36.2%), and high-risk (synchronous metastases with N1/N2 disease, 5-year OS 13.8%) (25), however this classification scheme is yet to be formally validated in clinical trials.

The number (and possibly volume) of metastatic sites has also been shown to be a potential prognostic factor. In a SWOG study by Albain et al., involving 2,531 patients with advanced NSCLC, median survival was highest in patients with a single lesion (8.7 months), compared to patients with multiple lesions in one organ (6.2 months) and multiple lesions in multiple organs (5.1 months) (26). Similarly in the subgroup analysis of RTOG 9508 trial, which allowed up to 3 brain metastases, survival improvement (with the addition of stereotactic radiosurgery) was only found in patients with a single lesion compared to 2–3 lesions (27). Looking at the use of SBRT in particular, patients with up to 3 lesions had a better OS compared to patients with 4–5 lesions (2-year OS 60.3 vs. 21.9%). However, it must be noted that only 11 of 61 patients had NSCLC (28).

## SBRT to Extra-cranial Sites Commonly Seen With Oligometastatic NSCLC (Lung, Liver, Adrenal)

- Lung: Prior studies on SBRT in primary NSCLC have reported local control rate comparable to surgery when the biologically effective dose (BED) of SBRT was at least 100 Gy (29–32). De Rose et al. reviewed 60 patients treated with SBRT for lung metastases in NSCLC with 60 Gy in 3 fractions to peripheral lesions <2 cm, 48 Gy in 4 fractions to peripheral lesions between 2 and 5 cm, and 60 Gy in 8 fractions to central lesions (30). All patients received a BED > 100 Gy resulting in a 2-year local control rate of 88.9% and 1- and 2-year OS of 94.5 and 74.6%, respectively. Laterality of metastatic disease does not seem to influence survival outcomes. For example, the survival was not significantly different between ipsilateral (T4, M0) vs. contralateral (M1a) surgical metastasectomy in 43 patients with NSCLC (27 vs. 43%) (33). Notably, none of the patients with mediastinal node involvement achieved long-term survival. More accurate staging with FDG-PET scan prior to SBRT significantly improved 1- and 2-year OS (82.7 vs. 72.8% and 64.8 vs. 52.6%, respectively, *P* = 0.012) (34). Pre-treatment performance status, maximum metastasis diameter, primary tumor histology, number of metastases, and time interval between primary tumor diagnosis and SBRT treatment significantly influenced OS (35). SBRT to the lung is generally well-tolerated with most patients experiencing grade 1–2 late pulmonary toxicity and grade 3 pulmonary toxicity in the minority (30, 31) and the BED at the planning target volume (PTV) isocenter was the only factor reported to influence toxicity in a database analysis of 700 patients treated with SBRT for oligometastatic lung disease (35).
- Liver: Ahmed et al. evaluated the radiosensitivity of liver metastases from different primary histology using a multigene expression index for tumor radiosensitivity (RSI) (36). They suggested that NSCLC has an intermediate radio-sensitivity (median RSI 0.31). Majority of the series reporting outcome of SBRT to liver metastases involve colorectal primaries. In the context of NSCLC, the presence of liver metastases has been associated with a worse prognosis compared to metastases to other sites in NSCLC (37, 38). Milano et al. evaluated the use of 50 Gy in 5 fractions for SBRT to treat hepatic metastases (~20% lung primary) and reported a 2-year local control rate of 67% (39). Rusthoven et al. (also ~20% lung primary) reported a higher 2-year local control rate of 92% with SBRT regimen of 30–60 Gy in 3 fractions (40). In a pooled analysis involving 474 patients with 623 liver metastases (with mainly colorectal and breast primary), increasing the maximum isocenter BED to >150 Gy EQD2Gy, increased 1- and 2-year control rate of treated lesions from 77–83% and 64–70%, respectively (41).
- Adrenal: SBRT to adrenal metastases in OM-NSCLC was specifically evaluated in a study by Celik et al. whereby 15 patients received 42 Gy in 6 fractions of CyberKnife® SBRT (42). One and two-year local control rates were 60 and 46.6%, respectively. Patient with metachronous metastases had a more favorable 2-year overall survival of 91.2% compared to 42.8% in patients with synchronous adrenal metastases. Holy et al. reported an overall median PFS of 4.2 months in their group of 18 patients with adrenal metastases from NSCLC treated with SBRT (range 20–40 Gy in 5 fractions) (43). Of these, 13 patients with isolated adrenal metastasis had longer median PFS of 12 months, local control rate of 77% (median follow-up: 21 months), and median OS of 23 months. SBRT for adrenal metastases is reasonably tolerated with previous studies reporting grade 1–2 toxicities including gastrointestinal toxicity, fatigue, rarely duodenal ulcers, and possibly late adrenal insufficiency (42, 44, 45).

## Summary of Evidence Supporting SBRT in OM-NSCLC

A retrospective analysis of patterns-of failure after first-line systemic therapy in 387 patients with NSCLC reported local progression as the predominant pattern-of failure and suggested that local consolidative therapy with SBRT to known sites of disease following systemic therapy to prolong the time to first progression (46). Since then, trials of patients with limited metastatic NSCLC treated with SBRT have demonstrated significant survival benefit in both first and second line settings (Table 1).

**Table 1:** Selected studies of SBRT treatment in oligometastatic NSCLC.

**References**	**Year**	**Patients (*n*)**	**Site of oligo-met**	***N***	**Dose (Gy/fraction)**	**Systemic therapy**	**Median follow-up (months)**	**Median PFS (months)**	**Median OS (months)**
**RETROSPECTIVE STUDIES**									
Inoue et al. (47)	2010	41^*^	Brain, lung, adrenal	<5	48/8 (adrenal) 35–60/4–8 (lung)	NA	20	3-year PFS 20%	24
Holy et al. (43)	2011	18	Adrenal	NA	20–40/5	Various	21	4.2 (all) 12 (1 met)	23 (1 met)
Hasselle et al. (48)	2012	25	Multiple	<5	24–70/3–20	Chemo or targeted therapy	14	7.6	22.7
De Rose et al. (30)	2016	60	Lung	<5	48–60/3–8	Chemo	28	32.2 (actuarial)	32.1 (actuarial)
Celik et al. (42)	2017	15	Adrenal	<5	42/6	Chemo	24	10.5	2-year OS 46.6%
**SINGLE ARM PROSPECTIVE TRIALS**									
Salama et al. (28)	2012	61^*^	Multiple	<5	24–48/3	Chemo	20.9	2-year PFS 22%	2-year OS 56.7%
De Ruysscher et al. (49)	2012	40	Multiple	<5	54/3^**^	Chemo	27.7	12.1	13.5
Collen et al. (50)	2014	26	Multiple	<5	50/10	Chemo	16.4	11.2	23
**RANDOMIZED PHASE II TRIALS**									
Gomez et al. (12)	2016	49	Multiple	<3	NR	Chemo	12.4	14.2 vs. 4.4	41.2 vs. 17
Iyengar et al. (11)	2018	29	Multiple	<5	21–37.5/1–5	Chemo	9.6	9.7 vs. 3.5	Not reached vs. 17
Palma et al. (13)	2019	99	Multiple	<5	35–60/3–8	Chemo	25	12 vs. 6	41 vs. 28

*N, number of oligometastatic lesions per patient; OS, overall survival; NR, data not reported; PFS, progression free survival*.

* *Various primary histology including NSCLC*.

** *Only one patient received SBRT*.

### Single Arm Prospective Trials

1. Collen et al. reported on 26 patients with synchronous OM-NSCLC patients with up to 5 metastases treated with SBRT (50 Gy in 10 fractions) (50). Notably, patients with uncontrolled primary tumors were eligible. The primary endpoint was complete metabolic response (CMR) on PET (3 months post-SBRT). Seventeen patients underwent SBRT after upfront chemotherapy, and the remaining underwent SBRT (to all sites) as primary treatment. Sixty percent of patients achieved metabolic response, with half of reaching CMR. The median PFS was 11.2 months, and median OS 23 months.
2. De Ruysscher et al. included 40 patients with synchronous OM-NSCLC (≤5 lesions) who were amenable for radical therapy to all tumor sites including the primary (surgery, stereotactic radiosurgery, fractionated RT to a dose of 60 Gy, and one patient received treatment with 54 Gy in 3 fractions of SBRT) (49). The vast majority had a single metastatic focus, and were treated with upfront chemotherapy, and approximately half had brain metastases. They report a median PFS of 12.1 months, and OS of 13.5 months. The inferior results compared to the Collen study may be related the larger proportion of patients with brain metastases in this cohort, or the use of conventionally fractionated RT.
3. Bauml et al. recently published their single-arm Phase II trial comprising of 51 patients with ≤4 lesions who completed locally ablative therapy to all sites, following which they were given pembrolizumab. They reported a median PFS of 19.1 months and 1-year OS of 90.9%. This is notably much improved compared to historical controls (51).

### Randomized Phase II Trials

1. Iyengar et al. then conducted a randomized phase II trial for 29 patients with NSCLC and up to 5 OM lesions. NSCLC who had achieved partial response or stable disease to first-line chemotherapy (11). EGFR/ALK positive patients were excluded. They were randomized to SBRT + maintenance chemotherapy vs. maintenance chemotherapy alone. The trial was stopped early due to significant improvements with the addition of SBRT (PFS 9.7 vs. 3.5 months, *P* = 0.01). Toxicities were similar in both arms.
2. Gomez et al. conducted a multi-center Phase II randomized study in 49 patients with up to 3 OM NSCLC with no progression for at least 3 months post 1st line chemotherapy (12, 52). Eighty-four percent were EGFR/ALK negative. Patients were assigned to local therapy (surgery or radical RT) vs. maintenance chemotherapy or observation. Like the previous trial, this study was stopped early due to significant improvements in PFS in the local therapy arm (PFS 14.2 vs. 4.4 months, *P* = 0.022). OS was also significantly improved (OS 41.2 vs. 17 months, *P* = 0.017). There are two observations from this study. Firstly, the OS benefit was seen despite patients crossing-over from maintenance/observation to local therapy, suggesting earlier local therapy to be superior to local therapy on progression. Secondly, none of the patients suffered from Grade 3 toxicity.
3. Palma et al. conducted the international SABR-COMET Phase II trial including 99 patients with up to 5 OM lesions from a variety of primary histological types (20% lung primary) (13). Patients were randomized to SBRT to all sites vs. palliative standard of care alone. The primary endpoint, which was OS, was prolonged with addition of SBRT (41 vs. 28 months, *P* = 0.09). Unfortunately, there were significantly more toxicity in the SBRT arm (29 vs. 9%) with treatment-related death (Grade 5) being experienced by three patients (4.5%).

### Phase III Trials

No Phase III trial has reported the benefit of SBRT in OM-NSCLC. In view of the convincing Phase II data, there are multiple ongoing Phase III trials which are eagerly awaited. These trials are summarized in Table 2.

**Table 2:** Selected ongoing trials of SBRT treatment in oligometastatic NSCLC.

**Title**	**Patients**	**Study design**	**Estimated completion**
Stereotactic Ablative Radiotherapy for Oligometastatic Non-small Cell Lung Cancer (SARON). A Randomized Phase III Trial. (53) Institution: University College London ClinicalTrials.gov identifier: NCT02417662	340	Phase 3 multi-center: chemotherapy alone (standard platinum based doublet chemotherapy or chemotherapy + radical radiotherapy (conventional RT and SABR) Primary histology: all NSCLC *1–3 oligometastatic lesions* Primary outcome measure: OS	August 2022
Maintenance Systemic Therapy vs. Local Consolidative Therapy (LCT) Plus Maintenance Systemic Therapy for Limited Metastatic Non-small Cell Lung Cancer (NSCLC): A Randomized Phase II/III Trial (NRG LU-002) Institution: NRG Oncology ClinicalTrials.gov identifier: NCT03137771	300	Phase 2/3 multi-center: maintenance chemotherapy or SBRT + maintenance chemotherapy Primary histology: all NSCLC *1–3 oligometastatic lesions* Primary outcome measure: PFS	April 2022
Randomized Phase III Trial of Local Consolidation Therapy (LCT) After Nivolumab and Ipilimumab for Immunotherapy-Naive Patients With Metastatic Non-small Cell Lung Cancer (LONESTAR) -Strategic Alliance: BMS Institution: M.D. Anderson Cancer Center ClinicalTrials.gov identifier: NCT03391869	270	Phase 3 multi-center: systemic treatment only with nivolumab and ipilimumab or induction nivolumab and ipilimumab followed by local consolidative therapy with surgery and/or radiotherapy Primary histology: all NSCLC *>1 oligometastatic lesions* Primary outcome: OS	December 2022
A Randomized Trial of Conventional Care vs. Radioablation (Stereotactic Body Radiotherapy) for Extracranial Oligometastases (CORE) Institution: Royal Marsden NHS Foundation Trust ClinicalTrials.gov identifier: NCT02759783	245	Phase 2/3 multi-center: standard of care or standard of care + SBRT Primary histology: breast, prostate, or NSCLC *1–3 oligometastatic lesions* Primary outcome measure: PFS	October 2024
A Randomized Phase III Trial of Stereotactic Ablative Radiotherapy for the Comprehensive Treatment of 4–10 Oligometastatic Tumors (SABR-COMET 10) Institution: Lawson Health Research Institute ClinicalTrials.gov identifier: NCT03721341	159	Phase 3 multi-center: stereotactic ablative radiotherapy, plus standard of care treatment: chemotherapy, immunotherapy, hormones, or observation given at the discretion of the treating oncologist Various histology including NSCLC *4 to 10 oligometastatic lesions* Primary outcome: OS	January 2029
Randomized Phase II Trial of Local Consolidation Therapy (LCT) After Osimertinib for Patients With EGFR Mutant Metastatic Non-small Cell Lung Cancer (NSCLC) (NORTHSTAR) Institution: M.D. Anderson Cancer Center ClinicalTrials.gov identifier: NCT03410043	143	Phase 2 multi-center: osimertinib followed by local consolidative therapy with surgery and/or radiotherapy or maintenance osimertinib alone Primary histology: NSCLC *>1 oligometastatic lesion* Primary outcome: PFS	January 2023
A Multicentre Single Arm Phase II Trial Assessing the Efficacy of Immunotherapy, Chemotherapy and Stereotactic Radiotherapy to Metastases Followed by Definitive Surgery or Radiotherapy to the Primary Tumor, in Patients With Synchronous Oligo-metastatic NSCLC Institution: European Thoracic Oncology Platform ClinicalTrials.gov identifier: NCT03965468	47	Phase 2 multi-center: durvalumab, carboplatin/paclitaxel chemotherapy, followed by SBRT to all oligometastases. Restaging at 3 months Definitive local treatment with surgical resection of primary tumor or RT 60–66 Gy to the primary tumor if no disease progression. *1–3 oligometastatic lesions* Primary outcome: PFS	December 2021

*RT, radiotherapy; SBRT, stereotactic body radiation therapy; SABR, stereotactic ablative radiotherapy; OS, overall survival; PFS, progression free survival*.

## Future Direction and Unanswered Questions

Considerable progress has been made in the realm of OM-NSCLC. Improvements in survival stem partly from more effective systemic therapy, but also aggressive consolidation therapies (surgery, radiation) in patients with a favorable disease biology. Although the results from randomized Phase II data are exciting, adequately powered Phase III trials with clear inclusion/exclusion criteria (e.g., synchronous, metachronous, oligorecurrence) and appropriate primary endpoints are much awaited to change practice. The upper limit of the number of acceptable OM lesions were set rather arbitrarily. It remains unclear if we should limit this to 3, 5 or 10 (54). As such, two randomized Phase III trials are being planned. SABR-COMET 3 (NCT03862911) for 1–3 lesions, and SABR-COMET 10 (NCT03721341), for 4–10 lesions. Moreover, most of the prospective OM-NSCLC trials have been performed in the Caucasian population where EGFR/ALK driver mutations are known to be much lower than in Asian countries. There remain many unanswered questions about how best to manage these patients including clinical uncertainty if these principles can be extrapolated to populations with higher prevalence of driver mutations. Lastly, most of the studies were conducted prior to the use of immunotherapy. Therefore, the role of SBRT in the context of immunotherapy is uncertain.

## Conclusion

Stage IV NSCLC represents a heterogenous group of patients with an overall poor outcome. However, a sub-group of patients with limited metastatic disease may achieve long-term survival with effective systemic therapy and aggressive local therapy. SBRT is a good option to obtain durable local control, and possibly prolong survival for these patients. At the same time, SBRT can be a double-edged sword, with toxicities in a minority of patients. As always, appropriate patient selection remains paramount, and ongoing Phase III trials will provide clarity.

## Author Contributions

CW, BV, and SL contributed conception and design of the study. CW and BV organized the database and wrote the first draft of the manuscript. All authors wrote sections of the manuscript, contributed to manuscript revision, read, and approved the submitted version.

### Conflict of Interest

SS reports grants from Varian Industries outside the submitted work. AL reports personal fees from Varian Medical Systems Inc. outside the submitted work. The Department of Radiation Oncology at VU University Medical Center has a research collaboration with Varian Medical Systems. BS has received honoraria and/or travel support from Varian Medical Systems and ViewRay. SL reports research support from Elekta AB in Elekta Gamma Knife ICON Expert Group outside of submitted work. The remaining authors declare that the research was conducted in the absence of any commercial or financial relationships that could be construed as a potential conflict of interest.
